# Nano-Patterned Magnetic Edges in CrGeTe_3_ for Quasi 1-D Spintronic Devices

**DOI:** 10.1021/acsanm.3c01008

**Published:** 2023-05-11

**Authors:** Avia Noah, Yishay Zur, Nofar Fridman, Sourabh Singh, Alon Gutfreund, Edwin Herrera, Atzmon Vakahi, Sergei Remennik, Martin Emile Huber, Snir Gazit, Hermann Suderow, Hadar Steinberg, Oded Millo, Yonathan Anahory

**Affiliations:** † The Racah Institute of Physics, The Hebrew University, Jerusalem 9190401, Israel; ‡ Laboratorio de Bajas Temperaturas, Unidad Asociada UAM/CSIC, Departamento de Física de la Materia Condensada, Instituto Nicolás Cabrera and Condensed Matter Physics Center (IFIMAC), Universidad Autónoma de Madrid, E-28049 Madrid, Spain; § Center for Nanoscience and Nanotechnology, Hebrew University of Jerusalem, Jerusalem 91904, Israel; ∥ Departments of Physics and Electrical Engineering, University of Colorado Denver, Denver, Colorado 80217, United States; ⊥ The Fritz Haber Research Center for Molecular Dynamics, The Hebrew University of Jerusalem, Jerusalem 91904, Israel

**Keywords:** nanomagnetism, edge magnetism, scanning SQUID microscopy, SQUID-on-tip, magnetic imaging, van der Waals ferromagnet, CrGeTe_3_

## Abstract

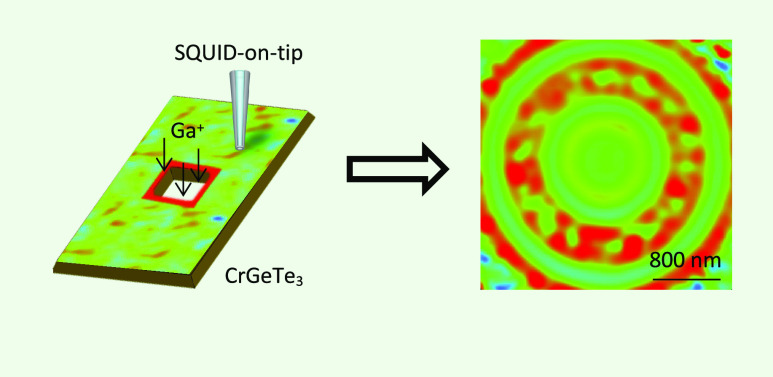

The synthesis of two-dimensional van der Waals magnets has paved the way for both technological applications and fundamental research on magnetism confined to ultra-small length scales. Edge magnetic moments in ferromagnets are expected to be less magnetized than in the sample interior because of the reduced amount of neighboring ferromagnetic spins at the sample edge. We recently demonstrated that CrGeTe_3_ (CGT) flakes thinner than 10 nm are hard ferromagnets; i.e., they exhibit an open hysteresis loop. In contrast, thicker flakes exhibit zero net remnant field in the interior, with hard ferromagnetism present only at the cleaved edges. This experimental observation suggests that a nontrivial interaction exists between the sample edge and the interior. Here, we demonstrate that artificial edges fabricated by focus ion beam etching also display hard ferromagnetism. This enables us to write magnetic nanowires in CGT directly and use this method to characterize the magnetic interaction between the interior and edge. The results indicate that the interior saturation and depolarization fields depend on the lateral dimensions of the sample. Most notably, the interior region between the edges of a sample narrower than 300 nm becomes a hard ferromagnet, suggesting an enhancement of the magnetic exchange induced by the proximity of the edges. Last, we find that the CGT regions amorphized by the gallium beam are nonmagnetic, which introduces a novel method to tune the local magnetic properties of CGT films, potentially enabling integration into spintronic devices.

## Introduction

Low-dimensional magnetism^1−3^ and specifically magnetically ordered van der Waals (vdW) materials^4−9^ have attracted much interest in recent years. The timely and essential progress made now enables the study of unconventional magnetic phenomena with no direct counterpart in bulk 3D materials. Some examples include quantum spin chains,^10−13^ magnetic nanoparticles,^14^ and two-dimensional magnetic layers.^15−20^ These provide promising options for the experimental realization of phenomena, such as quantum criticality^21^ and spin frustration,^22^ which have been the subject of numerous theoretical predictions. In particular, the intricate evolution of magnetic properties from bulk to thin exfoliated layers^9,23−25^ offers insights into the physical origin of ferromagnetism (FM) in vdW materials, where anisotropy is thought to be the result of distinct interlayer and intralayer exchange interactions.^4^

The vanishing remnant magnetization in zero field with increasing thickness is a phenomenon common to a number of vdW ferromagnetic materials.^25−27^ For example, thin CrGeTe_3_ (CGT) films (*d* < 10 nm) exhibit a net magnetization at zero applied field.^4,27^ In contrast, using SQUID-on-tip (SOT) microscopy and in situ magneto-transport measurements of CGT/NbSe_2_ bilayers, recent work demonstrated that the interior of thicker flakes (*d* > 10 nm) has zero remnant field, with hard FM appearing only at the sample edge.^27^ This CGT edge magnetization is confined to a magnetic nanowire with a width and thickness of a few tens of nanometers. However, the physical mechanism causing edge magnetism remains to be identified. Modulation of the edge shape by nanofabrication could provide information about the role of the geometry in edge magnetization and about its magnetic interaction with the sample interior.

Beyond the interest in finding the underlying physical mechanism, edge magnetism could be applied in spintronic devices where magnetic nanowires serve, for example, as racetrack memory devices.^28^ Here, we study edges nanofabricated by focused ion beam (FIB) and characterize them by scanning SOT microscopy.^29,30^ Our key result is that magnetic edges can be directly written using a FIB. This capability allows us to examine magnetic edge confinement and the magnetic interaction between the sample interior and the edge. Our results indicate that when two edges are closer than 300 nm, the interior becomes a hard ferromagnet. In addition, we demonstrate that CGT regions amorphized by the gallium beam are non-magnetic, which introduces a novel method to tune the local magnetic properties in CGT films.

## Results

### Directly Written Magnetic Edges

**Figure 1:**
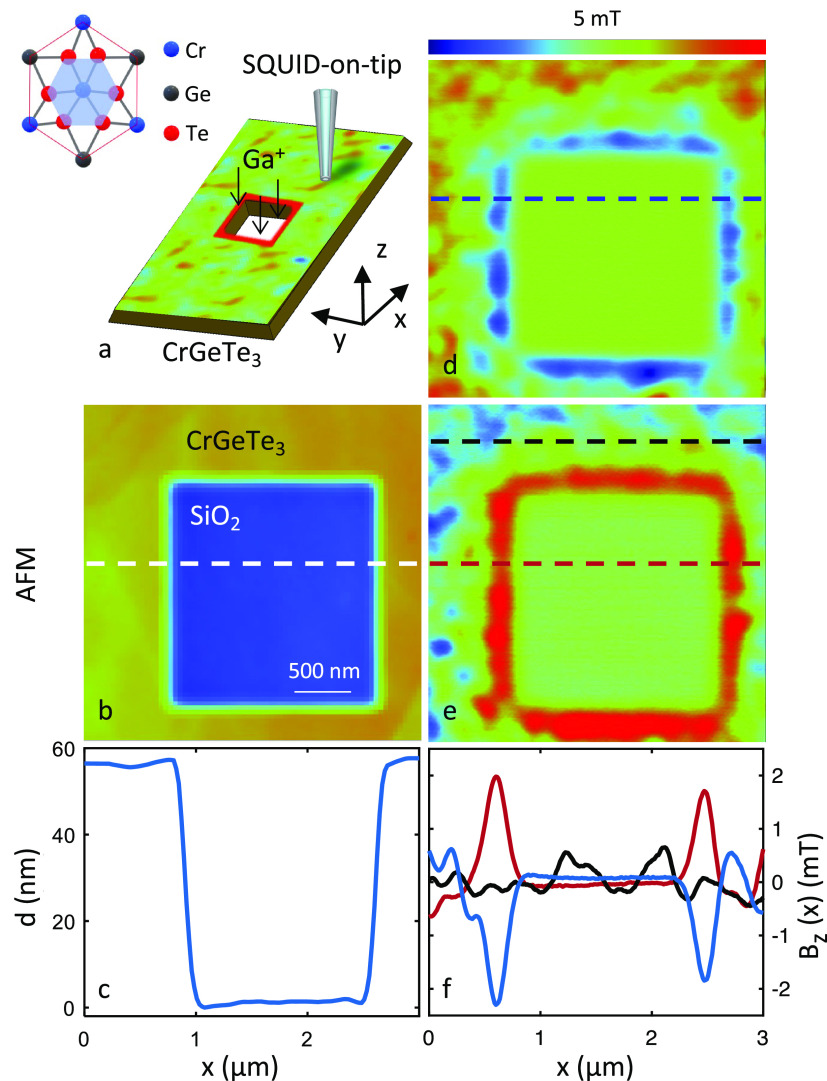
SQUID-on-tip (SOT) images of nano-patterned edges in CrGeTe_3_ at 4.2 K. (a) Schematic illustration of the measurement. (inset) Top view of the crystal structure. (b) Atomic force microscope (AFM) topographic image of the region of interest. (c) Topographic profile of the AFM measurement shown in (b). (d and e) *B_z_*(*x*, *y*) images acquired at μ_0_*H_z_* = 0 after opposite field excursion μ_0_*H_z_* = – 200 (d), 200 (e) mT. (**f**) Line profile of the magnetic signal along the *x*-axis containing edges (blue and red dashed lines) and only the interior (black dashed line). All images are 3 × 3 μm^2^ in size, pixel size 15 nm, and acquisition time 5 min/image. The blue to red color scale represents lower and higher magnetic fields, respectively, with a shared scale of *B_z_* = 5 mT.

In Figure 1a, we present a schematic illustration of the experimental setup. CGT flakes with thicknesses ranging from 50 to 110 nm were exfoliated on top of a SiO_2_-coated Si wafer. To create edges with controlled geometries, various shapes were etched out of the flakes using a Ga^+^ FIB. Local magnetic field imaging *B_z_*(*x*, *y*) with a scanning SOT at 4.2 K was used to characterize the magnetic properties of the edges and surrounding areas. We estimate the spatial resolution of our images to be approximately 150 nm (see Methods section and Supporting Note 1).

Figure 1b depicts the topography of a CGT 50 nm-thick flake from which a 2 × 2 μm^2^ square-shaped hole was etched. The corresponding topographic line profile presented in Figure 1c demonstrates that the CGT was completely removed from this region. The topographic data were acquired ex situ under ambient conditions with a commercial atomic force microscope (AFM). Figure 1d,e shows *B_z_*(*x*, *y*) images of the same area as Figure 1b. The field was ramped to μ_0_*H_z_* = – 200 mT for a few seconds and subsequently returned to μ_0_*H_z_* = 0 before the SOT images were acquired (Figure 1d). A similar field excursion was executed on a positive field (μ_0_*H_z_* = 200 mT) before the image shown in Figure 1e was acquired. In both images, we measured a net magnetic signal at the edge of the square. The direction of the measured field at the edge is negative/positive after the respective field excursions at the negative/positive applied magnetic field. In both cases, a disordered magnetic signal is observed ∼100 nm away from the edge. To further analyze these results, Figure 1f compares the magnetic signals cross-section *B_z_*(*x*) in locations indicated by dashed lines in Figure 1d,e. The peaks observed in *B_z_*(*x*) at the edges (Figure 1f blue and red curves) exhibit larger magnetic field values than the local field fluctuations in the disordered pattern observed far from the edges (Figure 1f black curve). Furthermore, at the edge, the field direction is determined by the field history, whereas the interior average magnetization vanishes. We note that the width of the magnetic edge is limited by our tip size (175 nm) and the magnetic edge is certainly sharper than shown in the *B_z_*(*x*) profile. Our results are consistent with the magnetism found at cleaved edges of exfoliated CGT,^27^ thereby substantiating the ability to write magnetic edges in arbitrary shapes.

### Amorphous CrGeTe_3_

**Figure 2:**
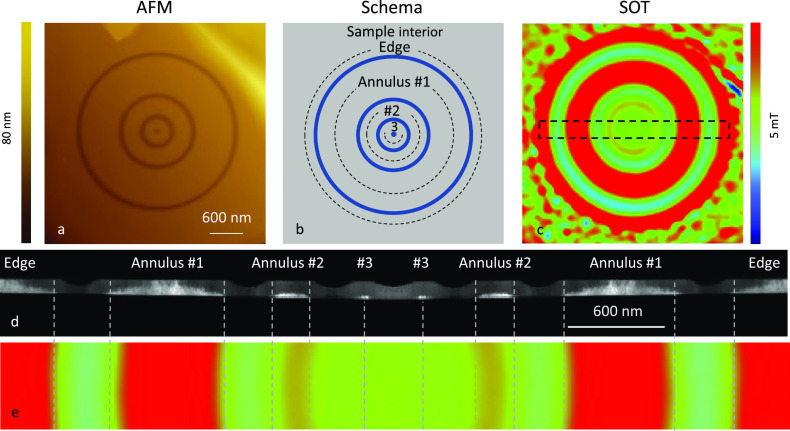
SOT images of nano-patterned annuli in CrGeTe_3_. (a) Atomic force microscope (AFM) image of CrGeTe_3_ (CGT) patterned using the focused ion beam (FIB) into annuli with outer diameters OD_1_ = 2800 nm, OD_2_ = 1200 nm, and OD_3_ = 350 nm. (b) Schema of the annuli. The amorphized rings are shown in blue. The three annuli and the outer edge are marked with dashed circles. (c) *B_z_*(*x*, *y*) images acquired at μ_0_*H_z_* = 0 after field excursion μ_0_*H_z_* = 200 mT. (d) Scanning transmission electron microscope (STEM) cross-sectional image measured along the black rectangle presented in (c). The crystalline CGT appears in white, while the amorphized CGT appears in dark gray. The image was symmetrized around its center for clarity. (e) SOT image corresponding to the rectangle in c and matching the STEM cross-section in d at μ_0_*H_z_* = 0 mT. The gray dashed lines are a visual guide to facilitate the correlation between the crystalline CGT in the STEM d and SOT e image. The images are 4.5 × 4.5 μm^2^ c and 3 × 0.5 μm^2^ e, pixel size 18 nm, acquisition time 5 min/image. The blue to red color scale represents lower and higher magnetic fields, respectively, with a shared scale of *B_z_* = 5 mT. The signal of annulus #1 intentionally saturates the color scale to allow the signal of annulus #2 to be visible on that scale.

To characterize all the possible effects of the FIB, we must also consider the potential amorphization of the CGT caused by the Ga^+^ ion beam. We therefore configure the FIB to obtain a partially etched pattern that consists of three concentric annuli with different outer diameters (OD_1_ = 2800 nm, OD_2_ = 1200 nm, OD_3_ = 350 nm) as depicted in the AFM image in Figure 2a and illustrated schematically in Figure 2b. The grooves visible in the AFM are not as deep as the thickness of the sample (∼20 nm < *d* = 50 nm). Figure 2c shows the *B_z_*(*x*, *y*) image corresponding to the same area as the AFM image shown in Figure 2a. To spatially resolve the resulting crystallographic structure, we prepared a scanning transmission electron microscopy (STEM) cross-section of the lamella corresponding to the region marked with the dashed line in Figure 2c (see the Methods section). The high-angle annular dark field (HAADF) image is shown in Figure 2d. The crystalline material appears brighter in the STEM than the amorphized region (see also Supplementary Note 3 and Figure S3). We note that the circles defining the annuli are not completely etched but that the remaining CGT is entirely amorphized. The area surrounding the etched area is also amorphized due to the finite beam size effect. As a result, the CGT crystals are embedded in the amorphous material. We also note that annulus #3 is almost completely amorphized, while the other annuli retain a significant amount of crystalline material.

The *B_z_*(*x*, *y*) image (Figure 2c) was acquired at μ_0_*H_z_* = 0 after a field excursion of μ_0_*H_z_* = 200 mT. We describe the magnetic features of this image starting from the frame of the image and going toward the center. In the region far from the annuli, the SOT image reveals the disordered magnetic domains averaging to zero magnetization. Next, we observe a ring color-coded in red, which corresponds to the edge of the sample interior in the vicinity of the largest amorphized ring. The amorphized ring appears in our SOT image as green and light blue. The annuli appear toward the center of the image. Figure 2e presents the annuli stray field as a zoomed-in *B_z_*(*x*, *y*) image corresponding to the region marked in Figure 2c and matching the region of the STEM cross-section in Figure 2d. The outer red color-coded ring corresponds to annulus #1, which is fully magnetized at the zero applied field. Further inward is a smaller green color-coded ring, which is nonmagnetic and corresponds to the etched region between annuli #1 and #2. Annulus #2 appears in the SOT image as a softer red color-coded ring. We note that annulus #3 is nonmagnetic as the central area is color-coded in green at all measured applied fields.

By correlating the SOT *B_z_*(*x*, *y*) and STEM images, we conclude that all the amorphized regions, which include the three etched rings and annulus #3, are nonmagnetic. Thus, we can define effective crystalline dimensions, for the width and thickness, *w*_e_ and *d*_e_. The effective crystalline dimensions of the outer annulus #1 are *w*_e_ = 500 nm and *d*_e_ = 40 nm, and those of the middle annulus #2 are *w*_e_ = 100 nm and *d*_e_ = 10 nm, which are comparable with the CGT domain size. This confinement gives rise to two distinct magnetic properties around the coercive field (*H*_c_). At *H_z_* ∼ *H*_c_, the annulus #1 is large enough to break into magnetic domains, while annulus #2 remains a single domain and the magnetization of the entire area reverses abruptly (See Supporting Note 2).

### Edge-Interior Magnetic Interaction

**Figure 3:**
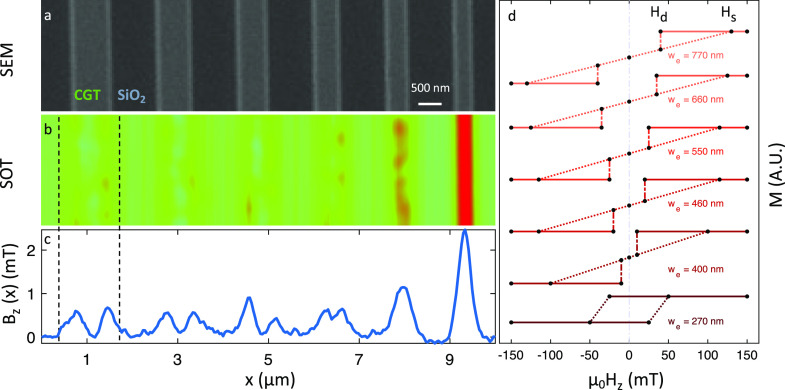
From 2-D to 1-D magnetic stripes. (a) Scanning electron microscopy (SEM) image of CrGeTe_3_ (CGT) with effective thickness *d*_e_ = 50 nm patterned into stripes with varying effective widths (*w*_e_) and length of 10 μm. (b) SQUID-on-tip (SOT) magnetic image *B_z_*(*x*, *y*) acquired at μ_0_*H_z_* = 0 after positive field excursion to μ_0_*H_z_* = 200 mT. For stripes with *w*_e_ > *w*_c_, two distinct magnetized edges (red color scale) separated by a zero average magnetization in the stripe’s interior (color-coded in green). For stripes with width *w*_e_ < *w*_c_ (right stripe), the two edges appear to merge and form a single magnetic domain. (c) Line profile of the magnetic signal along the *x*-axis of the image in (b). The line profile was averaged over 45 pixels. (d) Sketched magnetization curves drawn from *B_z_*(*x*, *y*) acquired on stripes with different widths. Dashed lines are a guide to the eye connecting the saturated fields (*H*_s_) and the demagnetization field (*H*_d_). The fields at which the images were taken are marked with black dots. The SOT image is 2.5 × 10 μm^2^, pixel size 40 nm, and acquisition time 5 min/image. The blue-to-red color scale represents lower and higher magnetic fields, respectively, with a scale of *B_z_* = 5 mT.

In the next section, we use our ability to control nanoscale magnetic patterns in CGT to make a systematic study of the magnetic properties as a function of lateral dimensions. For this purpose, we use the Ga^+^ FIB to fabricate 10 μm long CGT stripes with varying widths (*w*_e_), as presented in the SEM image in Figure 3a. Figure 3b presents a *B_z_*(*x*, *y*) image of the stripes acquired at μ_0_*H_z_* = 0 after a field excursion at μ_0_*H_z_* = 200 mT. The images of the wider stripes include two distinct magnetized edges (color-coded in red) separated by a zero-average magnetization in the stripe’s interior (color-coded in green). However, below a certain critical width *w*_c_ ∼ 300 nm, these two edges appear to merge to form a single magnetic domain. Figure 3c presents a line profile of the image shown in Figure 3b along the *x*-axis (*B_z_*(*x*)). We note that the *B_z_*(*x*) signal for the narrow stripe is four times larger than the signal at a single edge. This finding suggests that the stripe interior also becomes a hard ferromagnet because of the edge proximity.

**Figure 4:**
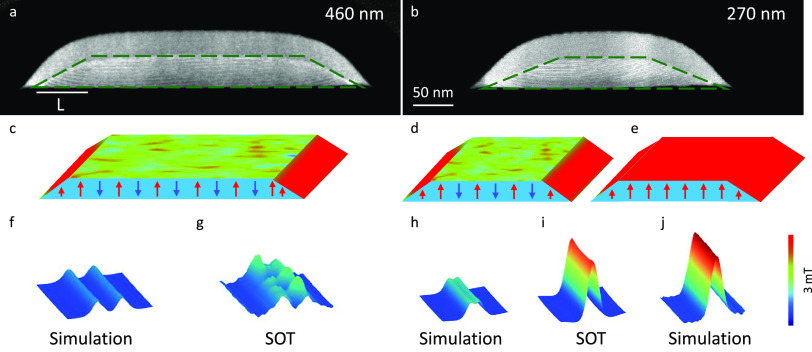
STEM images of the CrGeTe_3_ stripes and magnetostatic simulations. (a and b) Scanning transmission electron microscope (STEM) cross-sectional images measured in the middle of the stripe with effective widths *w*_e_ = 460 nm a, and *w*_e_ = 270 nm b. The effective crystalline CrGeTe_3_ is marked with green dashed lines. (c–e) Schematic illustration of the local magnetic structure at the edges and interior for different stripe widths. In panels c and d, the edges but not the interior of the sample retain their magnetization. In panel e, the whole stripe is a hard ferromagnet. (f–j) Comparison between the SQUID-on-tip (SOT) images and magnetostatic simulations. (f, h) Simulations of the stripe magnetization resulting from the magnetized edges for a triangular cross-section, marked by red in panels c and d. (j) Simulations of the stripe magnetization assuming that the whole stripe is magnetized, marked by red in panel e. (g and i) *B_z_*(*x*, *y*) SOT images of the stripes in a and b. The blue to red color scale represents lower and higher magnetic fields, respectively, with a shared scale of 3 mT.

To understand this observation quantitatively, we carried out magnetostatic simulations assuming a magnetization of 3 μ_B_/Cr and a unit cell volume of 0.83 nm^3^.^31^ The tip-to-sample distance (170 nm) can be obtained by coupling the tip to a tuning fork to sense the surface.^32^ The geometry of the sample is obtained from the HAADF images considering that the amorphous CGT is nonmagnetic (Figure 4a,b). The stripe cross-section is trapezoid-shaped and marked with green dashed lines. Given this geometry, we define the effective width as . The STEM resolves an effective dimension of *w*_e_ = 460 nm (Figure 4a) and 270 nm (Figure 4b) with an effective thickness *d*_e_ = 50 nm.

For a wider stripe (*w*_e_ = 460 nm, Figure 4a), we model the edge by assuming a right-angled triangle cross-section with area *L* × *d*/2 = 2000 nm^2^ (Figure 4a). The simulated field distribution emanating from such a triangular cross-section edge (Figure 4f) is in good agreement with the measured SOT image (Figure 4g). Thus, the simulation confirms a magnetic edge width of a few tens of nanometers. The minor discrepancy observed between the SOT image and simulation may be due to local variations in the edge roughness or may be a consequence of the influence of the magnetic domains present in the bulk. Figure 4d presents the same calculation executed for the narrow stripe (*w*_e_ = 270 nm), by modeling the edges as a triangle cross-section of *L* × *d*/2 = 2500 nm^2^. In contrast to the wider stripe, this yields a poor agreement between the simulation (Figure 4h) and the SOT results (Figure 4i), where the simulated signal magnitude is smaller than the experimental data by a factor of four. To obtain good agreement, we need to assume that the entire stripe is magnetized as illustrated in Figure 4e and simulated in Figure 4j. We therefore conclude that the edges enhance the exchange interaction in the sample interior, resulting in a proximity-induced hard ferromagnetic state. The typical decay length of such interaction can be estimated as about *w*_c_ = 300 nm for *d*_e_ = 50 nm and this defines the critical width *w*_c_ for the emergence of hard FM in the sample interior.

The evolution of the magnetic profile seen in Figure 3b,c suggests that there is an abrupt transition from soft to hard ferromagnet in the sample interior. However, more precise magnetic characterization reveals that the transition is gradual. By measuring *B_z_*(*x*, *y*) as a function of the applied field *H_z_*, we can extract the width dependence of the saturation (*H*_s_) and demagnetization (*H*_d_) fields on the sample interior. Figure 3d summarizes the values of *H*_s_ and *H*_d_ found for each stripe, with the magnetization hysteresis curves marked as a dashed line connecting the dots corresponding to *H*_s_ and *H*_d_ as a visual guide. For *w* > *w*_c_, *H*_d_ and *H*_s_ grow with *w*, which produces a bowtie hysteresis curve with no remnant field in the sample interior. For *w* < *w*_c_, the demagnetization field crosses zero and becomes the coercive field *H*_c_, which results in an open hysteresis loop. In this case, the stripe breaks into magnetic domains at the coercive field μ_0_*H_c_* = ± 25 mT and the magnetization saturates at μ_0_*H*_s_ = ± 50 mT. This set of measurements indicates that the continuous effect of magnetic confinement before the transition causes the sample interior to become a hard ferromagnet. It is important to note that for *w* < *w*_c_, the sample interior is still sufficiently large to accommodate magnetic domains at the coercive field. Thus, the observed transition does not coincide with a single-domain transition although *w*_c_ = 300 nm is comparable with the magnetic domain size (∼100 nm).

## Discussion

One plausible underlying physical mechanism for edge magnetism is related to the wedge shape seen in cleaved edges.^27^ Given that thin flakes (*d* < 10 nm) are hard ferromagnets and considering that part of the wedge must be thinner than 10 nm, this mechanism appears relevant for cleaved edges. Since etched edges possess a similar angle that obtained upon cleaving (20° to 30°), the same mechanism could also explain edge magnetism in etched edges.

Another potential explanation for edge magnetism in etched edges could be the Ga^+^ contamination. Cross-sectional and energy-dispersive X-ray spectroscopy (EDS) measurements of the annuli (Figure 2) and the stripes (Figure 3) addressing the Ga contamination and CGT oxidation are presented in Supporting Figures S4–6. Importantly, in the crystalline regions, the Ga concentration is uniform and below the background level ∼1.5 at %. The highest Ga concentration (15 at %) is uniformly distributed on the surface of the amorphized region where the concentration is 2.5 at %. We note that regions with the highest Ga concentration are amorphized and were found to be nonmagnetic. This observation suggests that if the presence of Ga has an effect, it would be to hinder magnetism rather than enhancing it. We note that the uniformity of the Ga distribution suggests that the edge magnetism cannot be explained by the Ga concentration profile. Oxygen contamination is spread uniformly near the CGT surface. No measurable amount of oxygen was observed 5 nm below the surface.

Strain should also be considered as one of the mechanisms to induce edge magnetization. It was shown that strain can enhance magnetism in CGT.^33,34^ It is plausible that some strain appears at low temperatures at the interface between the amorphous and crystalline CGT regions. The last mechanism that we can consider is related to the in-plane dangling bond. This mechanism was previously excluded^27^ since no magnetic edge was found at the step edge between two terraces of a single flake where in-plane dangling bonds would be expected. In addition, for nanofabricated edges, the magnetic edge is embedded in amorphous CGT, which should reduce the number of in-plane dangling bonds. Since the edge magnetism observed for these embedded edges is similar to that of an exfoliated sample, we consider this mechanism to be unlikely.

## Conclusions

In conclusion, we have demonstrated that quasi-1D magnetic edges can be directly written to form arbitrary shapes by using the FIB. That capability allows us to measure the effect of lateral confinement. In particular, we have shown that when two edges are separated by less than 300 nm, the whole sample becomes a hard ferromagnet. This suggests that geometry can influence the microscopic exchange interaction, strengthening ferromagnetic exchange over large distances. In addition, we report that an amorphous CGT material is nonmagnetic, which introduces an additional method to control the local magnetism. The directly written magnetic structure could be useful in devices that require very narrow magnetic channels, and we believe that the new method will have great potential for applications and fundamental research in confined magnetism and serve as a building block for spintronic devices.

## Methods

### Sample Fabrication

CrGeTe_3_ (CGT) crystals were grown using the flux method.^35^ CGT samples were fabricated using the dry transfer technique, which was carried out in a glovebox with an argon atmosphere. The CGT flakes were cleaved using the scotch tape method and exfoliated on commercially available Gelfilm from Gelpack.^27^ For the SQUID-On-Tip (SOT) measurements, a CGT flake was transferred to a SiO_2_ substrate. The various shapes were etched out of the CGT flakes with a Ga^+^ focused ion beam (FIB). The flakes were ∼50–110 nm thick as determined by atomic force microscopy and STEM measurements.

To fully etch the 110 nm-thick CGT flake as shown in Figure 3, we utilized a Ga^+^ ion beam operating at 30 kV and a 790 pA current. We etched a rectangular area of 10 μm^2^ during 10 s, which results in a fluence of ∼10^17^ Ga^+^/cm^2^. For the under-etched regions, such as the annuli rings shown in Figure 2, we employed a Ga^+^ ion beam at 30 kV and a 1.1 pA current. The largest ring had a diameter of 3 μm, and its width was estimated to the Ga beam profile (200 nm). In this case, we etched an area of 0.2 × 2π × 1.5 = ∼ 1.9 μm^2^ for 10 s, which resulted in a fluence of ∼10^15^ Ga^+^/cm^2^. To fully etch the 50 nm-thick CGT flake as shown in Figure 1, we utilized a Ga^+^ ion beam at 30 kV and a 7.7 pA current. We etched a square area of 4 μm^2^ for 18 s, which resulted in a fluence of ∼10^16^ Ga^+^/cm^2^.

### Scanning SQUID-on-Tip Microscopy

The SOT was fabricated using self-aligned three-step thermal deposition of Pb at cryogenic temperatures, as described previously.^29^ The measurements were performed using tips with effective SQUID loop diameters ranging from 145 to 175 nm. Figure S1 shows the measured quantum interference pattern of one of the SOTs used for this work, which has an effective diameter of 145 nm and a maximum critical current of 110 μA. The asymmetric structure of the SOT gives rise to a slight shift of the interference pattern, resulting in good sensitivity in zero fields. All measurements were performed at 4.2 K in a low-pressure He (between 1 and 10 mbar). All images were acquired with a constant distance between the tip and sample (170 nm). Under these conditions, the magnetic signal measured, which is on the order of 0.5–3 mT, is much larger than any possible parasitic influence related to the varying topography. That parasitic signal is estimated to be much smaller than our magnetic signal (<0.01 mT).

### Sample Characterization

High-resolution scanning electron microscope (SEM) cross-section lamellas were prepared and imaged by Helios Nanolab 460F1 Lite FIB—Thermo Fisher Scientific. The site-specific thin lamella was extracted from the CGT patterns using FIB lift-out techniques.^36^ STEM and Energy-Dispersive X-ray Spectroscopy (EDS) analyses were conducted using an Aberration Prob-Corrected S/TEM Themis Z G3 (Thermo Fisher Scientific) operated at 300 KV and equipped with a high-angle annular dark field detector (Fischione Instruments) and a Super-X EDS detection system (Thermo Fisher Scientific).

## Supporting Information Available

Quantum interference pattern of the SQUID-on-tip (Figure S1); SOT images of patterned annuli (Figure S2); zoomed-in STEM image of the 270 nm stripe (Figure S3); Cr, O, and Ga EDS line-scan of stripes (Figure S4); O, and Ga EDS maps of stripes (Figure S5); O, and Ga EDS maps of annuli (Figure S6); SOT fabrication and characterization (Supporting Note 1); SOT images of patterned annuli (Supporting Note 2); characterization of the amorphous and crystalline regions (Supporting Note 3) (PDF)

## List of Abbreviations

AFM: atomic force microscopy
*H*_c_: coercive field
*w*_c_: critical width
CGT: CrGeTe_3_
*H*_d_: demagnetization field
EDS: energy-dispersive X-ray spectroscopy
*d*_e_: effective thickness
*w*_e_: effective width
FM: ferromagnetism
FIB: focused ion beam
HAADF: high-angle annular dark field
OD: outer diameter
*H*_s_: saturation field
STEM: scanning transmission electron microscopy
SOT: SQUID-on-tip
*d*: thickness
vdW: van der Waals
