# Supplementary material for: Nano-Patterned Magnetic Edges in CrGeTe3 for Quasi 1-D Spintronic Devices
Source: ACS Appl Nano Mater. 2023 May 11;6(10):8627–34. doi: 10.1021/acsanm.3c01008 (PMC10226043; doi:10.1021/acsanm.3c01008)
Supplement: Supplementary file 1 — an3c01008_si_001.pdf [file an3c01008_si_001.pdf]

Supporting Information

Nano-Patterned Magnetic Edges in $CrGeTe_3$ for Quasi 1-D Spintronic Devices

Avia Noah[1], Yishay Zur[1], Nofar Fridman[1], Sourabh Singh[1], Alon Gutfreund[1], Edwin Herrera[2], Atzmon Vakahi[3], Sergei Remennik[3], Martin Emile Huber[4], Snir Gazit[1,5], Hermann Suderow[2], Hadar Steinberg[1], Oded Millo[1], and Yonathan Anahory*[1]*

[1]The Racah Institute of Physics, The Hebrew University, Jerusalem, 91904, Israel

[2]Laboratorio de Bajas Temperaturas, Unidad Asociada UAM/CSIC, Departamento de Física de la Materia Condensada, Instituto Nicolás Cabrera and Condensed Matter Physics Center (IFIMAC), Universidad Autónoma de Madrid, E-28049 Madrid, Spain

[3]Center for Nanoscience and Nanotechnology, Hebrew University of Jerusalem, Jerusalem, 91904, Israel

[4]Departments of Physics and Electrical Engineering, University of Colorado Denver, Denver, CO 80217, USA

[5]The Fritz Haber Research Center for Molecular Dynamics, The Hebrew University of Jerusalem, Jerusalem 91904, Israel

Email: avia.noah@mail.huji.ac.il, yonathan.anahory@mail.huji.ac.il

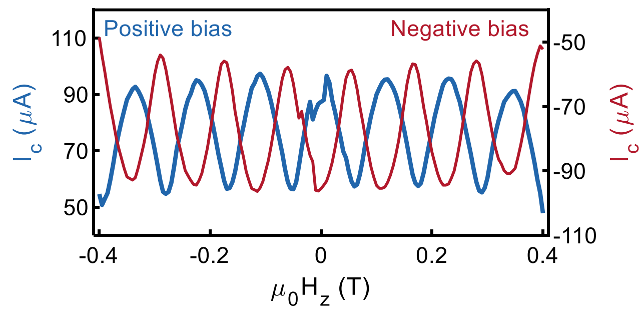

Figure S1. Quantum interference pattern of the SQUID-on-tip (SOT). The critical current I_c of one of the SOT's used in this work as a function of the applied out-of-Plane field H_z. Blue: Positive bias, red: Negative bias. The period of 120 mT of the quantum interference corresponds to an effective diameter of 145 nm of the SOT.

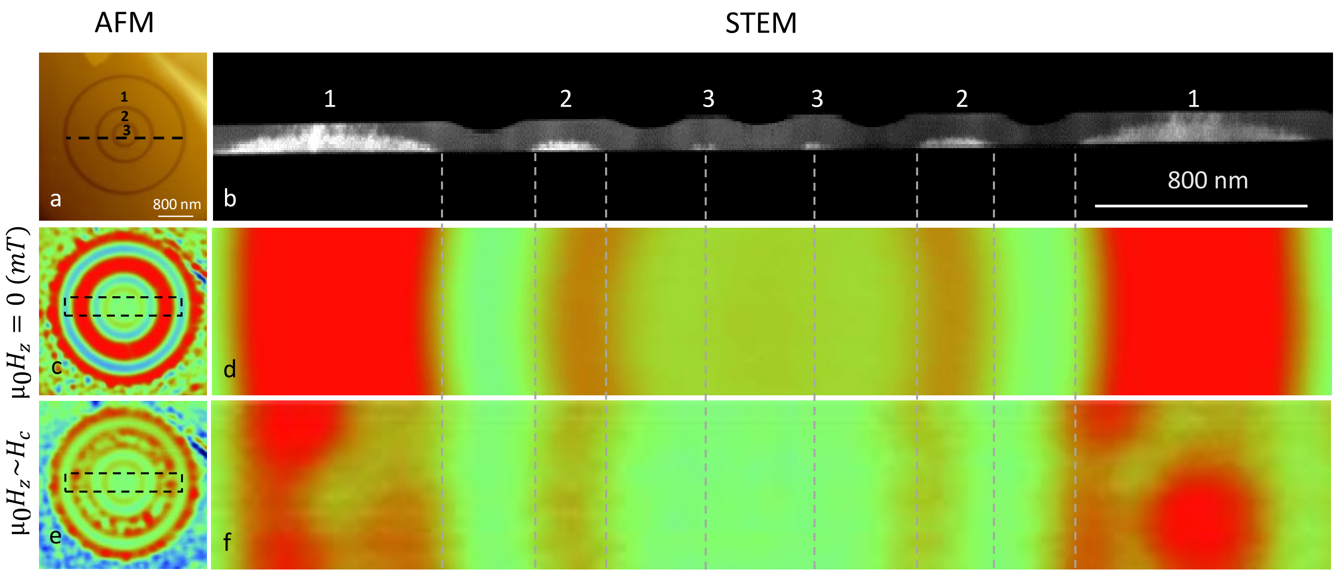

Figure S2. SOT images of patterned annuli in $CrGeTe_3$. (**a**) AFM image of $CrGeTe_3$ patterned using FIB into annuli with outer diameters $OD_1 = 2800$ nm, $OD_2 = 1200$ nm and $OD_3 = 350$ nm. (**b**) HAADF STEM cross-sectional image measured along the black line presented in **a**. The crystalline CGT appears in white, while the amorphized CGT appears in dark gray. (**c**) $B_z(x, y)$ images acquired at $\mu_0 H_z = 0$ after field excursion $\mu_0 H_z = 200$ mT. (**d**) SOT image corresponding to the rectangle in **c** and matching the STEM cross-section in **b**. (**e**) $B_z(x, y)$ images acquired at $0 < \mu_0 H_z < \mu_0 H_c$. (**f**) SOT image corresponding to the rectangle in **e**. At $H_z \sim H_c$, the annulus #1 is break into magnetic domains, while annulus #2 stays as a single domain. The dashed lines are a guide to the eye of the crystalline annuli. The images are 4.5×4.5 μm² **c,e** and 3×0.5 μm² **d**,**f**, pixel size 18 nm, acquisition time 5 min/image. The blue to red color scale represents lower and higher magnetic field, respectively, with a shared scale of $B_z = 5$ mT.

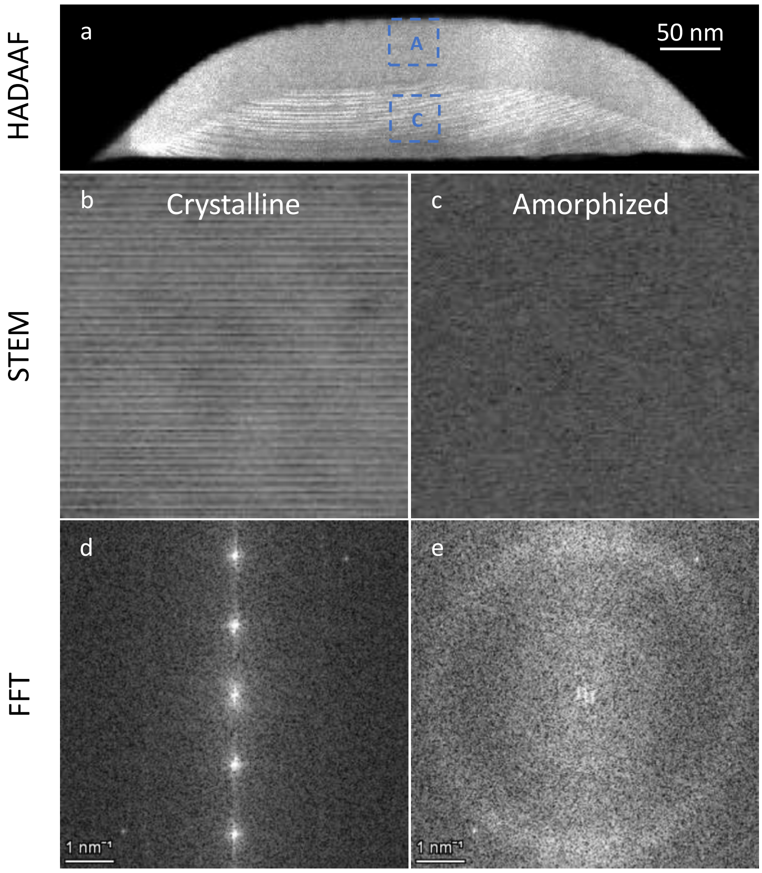

Figure S3. Zoomed-in STEM image of the 270 nm stripe in figure 4. (a) High-angle annular dark field (HAADF) image of the stripe cross section. **(b-c)** Zoomed-in of the crystalline region – bottom rectangle in **a** (b) and the amorphized region – top rectangle in **a** (c). **(d-e)** FFT of the images in b-c respectively, showing a Fourier transform of a crystal structure along the c axis (d) and of an amorphized structure (e).

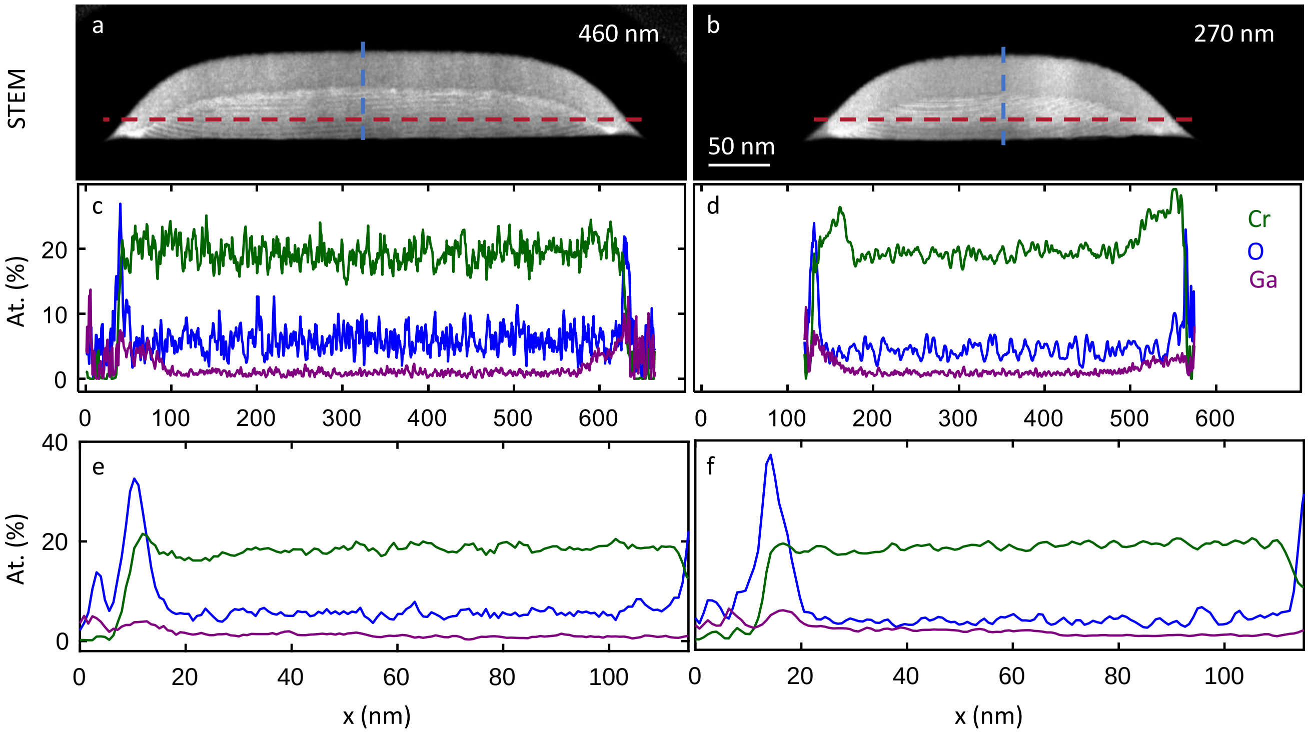

Figure S4. Cr, O, and Ga EDS line-scan of stripes in figure 4. (a-b) High-angle annular dark field (HAADF) image of the stripe cross section. **(c-f)** Energy-Dispersive X-ray Spectroscopy (EDS) line scan, showing the relative amount of Cr, O, and Ga in a horizontal (c,d) and vertical (e,f) cross sections of the stripes, in atomic percent.

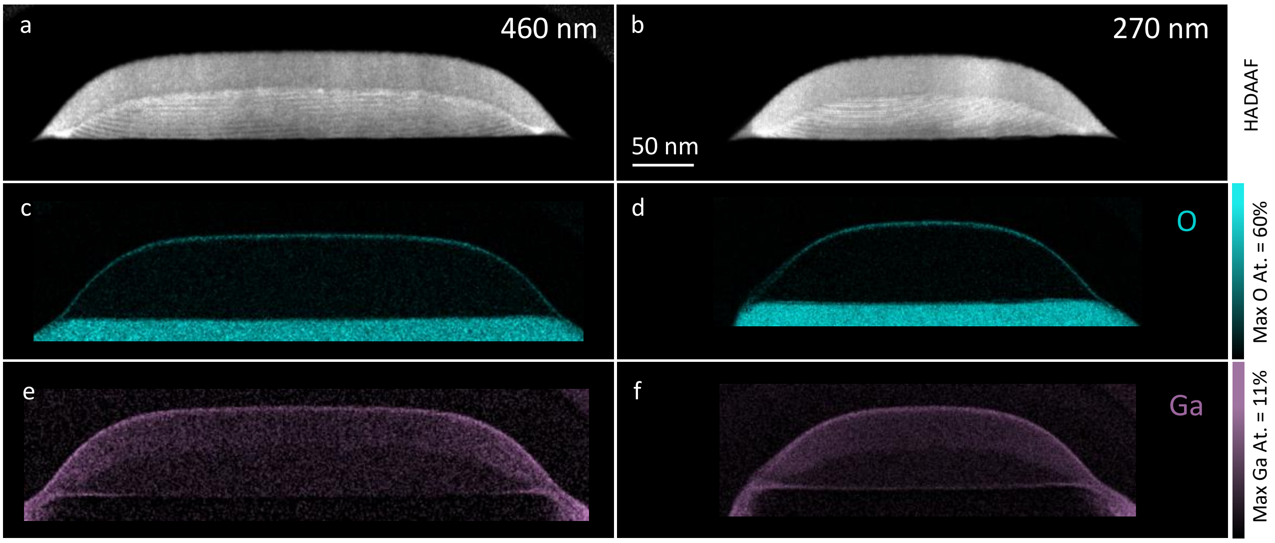

Figure S5. O, and Ga EDS maps of stripes in figure 4. (a-b) High-angle annular dark field (HAADF) image of the stripe cross section. **(c-f)** Energy-Dispersive X-ray Spectroscopy (EDS) map, showing the relative amount of O (c,d), and Ga (e,f) in a cross sections of the stripes, in atomic percent.

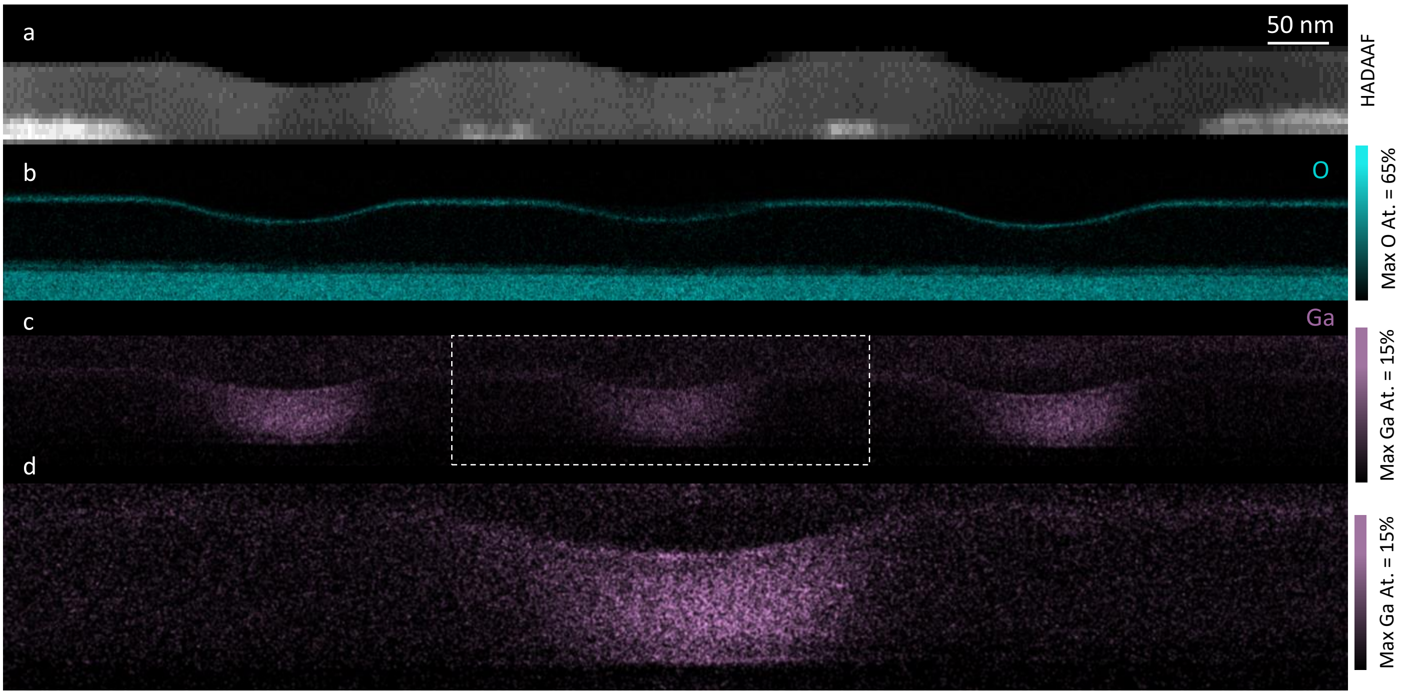

Figure S6. O, and Ga EDS maps of annuli in figure 2. (a) High-angle annular dark field (HAADF) image of the center annuli cross section. **(b-d)** Energy-Dispersive X-ray Spectroscopy (EDS) line scan, showing the relative amount of O (b) and Ga (c-d) in a cross section of the annuli, in atomic percent. (**d**) Zoomed-in image of the dashed rectangle in c.

Supporting Note 1: SOT fabrication and characterization

Figure S1 shows the quantum interference pattern typical for a SQUID. The SOT response to magnetic field is defined as the derivative of that pattern. To get good images, the SOT has to be field biased in a region where the interference pattern is linear, and one must avoid the regions where the response is zero (blind spots). The field period of this pattern is determined by the SQUID loop diameter so that each period represents a magnetic flux equal to the quantum of flux $\phi_0 = h/2e \approx 20.67$ Gµm^2. This means that a smaller SQUID loop will exhibit a large field period with larger linear regions but with larger blind spots. Depending on the experimental requirements, optimal SQUID loop size is chosen. To mitigate the effect of blind spots, we use the fact that our SOTs are often composed of asymmetric junctions. This makes the interference pattern to have a different field offset for a different direction of the current running through the SQUID (Figure S1, blue and red curves).

The magnetic length scale in $CrGeTe_3$ is on the order 100 nm or smaller. That implies a SQUID loop with a diameter below 100 nm. However, having a SQUID with a loop too small, of 50 nm for example, would yield a blind spot of +/- 0.4 T around zero field. That would be problematic to get good images below the saturation field (0.13 T). For this reason, all the SOTs used in this work have a SQUID loop of about 145 to 175 nm.

Supporting Note 2: SOT images of patterned annuli in $CrGeTe_3$

Here we compare between the annuli magnetizations. As mentioned in the manuscript, the CGT sample were partially etched using FIB and consists of three concentric annuli with different outer diameters (OD_1 = 2800 nm, OD_2 = 1200 nm, OD_3 = 350 nm) as depicted in the AFM image (Figure S2a). The STEM cross-section lamella in Figure S2b corresponding to the region marked with the dashed line in Figure S2a. Figure S2c shows the $B_z(x,y)$ image corresponding to the same area as the AFM image. The image was acquired at $\mu_0 H_z = 0$ after field excursion of $\mu_0 H_z = 200$ mT. To describe the annuli magnetization, we present in Figure S2d a zoomed-in $B_z(x,y)$ image corresponding to the region marked in Figure S2c. The red color-coded ring corresponds to annulus #1, which is fully magnetized at zero applied field. The next to that feature is a smaller nearly green color-coded ring, which is non-magnetic. This corresponds to the region between annulus #1 and #2. Annulus #2 is then visible as softer red color-coded. We note that annulus #3 is non-magnetic as the central area is green color-coded at all measured applied fields.

Now, we discuss the difference between the magnetic properties of annulus #1 and #2. The effective crystalline dimensions of annulus #1 are $w_e = 500$ nm and $d_e = 40$ nm. For annulus #2 the dimensions are $w_e = 100$ nm and $d_e = 10$ nm. This confinement gives rise to two distinct magnetic properties around the coercive field. In Figure S2e, we present the $B_z(x,y)$ image corresponding to the same area as Figure S2c but at $H_z \sim H_c$. In Figure S2f we present a zoomed-in $B_z(x,y)$ image of the marked area (same area as Figure S2d). Annulus #1 and annulus #2 hold the magnetization at zero field. However, at $H_z \sim H_c$, the annulus #1 is sufficiently large to break into magnetic domains, while annulus #2 stays as a single domain, and the magnetization of the entire area reverses abruptly. We attribute the single domain effect to the lack of space to accommodate magnetic domains.

Supporting Note 3: Characterization of the amorphous and crystalline regions.

To distinguish between the crystalline and amorphized regions we present in figure S3 high-magnification images of the 270 nm STEM image with the corresponding FFT analysis. In the crystalline part, the atomic layers are clearly resolved as seen in pristine samples and the FFT peaks corresponding to the interatomic layer are visible. In the amorphous region, we see a circular pattern in the FFT in agreement with an amorphous phase.
